# Exploring the use of self-sovereign identity for event ticketing systems

**DOI:** 10.1007/s12525-022-00573-9

**Published:** 2022-07-30

**Authors:** Simon Feulner, Johannes Sedlmeir, Vincent Schlatt, Nils Urbach

**Affiliations:** 1 Branch Business & Information Systems Engineering, Fraunhofer Institute for Applied Information Technology FIT, Wittelsbacherring 10, 95447 Bayreuth, Germany; 2 FIM Research Center, University of Bayreuth, Wittelsbacherring 10, 95447 Bayreuth, Germany; 3 ditlab, Frankfurt University of Applied Sciences, Hungener Straße 6, 60389 Frankfurt, Germany

**Keywords:** Bot prevention, Digital identity management, Digital wallet, Secondary market control, Ticket scalping, Verifiable credentials, O 14

## Abstract

Ticket fraud and ticket scalping activities often cause high costs as well as trust concerns for fans buying event tickets, especially in the secondary ticketing market. To address these issues, several publications and projects have proposed using blockchain technology to enable digital trust and ticket verifiability and thus to improve event ticketing systems. However, these approaches exhibit considerable privacy challenges and fall short concerning reliable, efficient visitor identification, which is necessary for controlling secondary market transactions. We demonstrate how a novel paradigm for end-user digital identity management, called self-sovereign identity (SSI), can be utilized to gain secondary market control. To do so, we follow a rigorous design science research approach to build and evaluate an SSI-based event ticketing framework. Our findings demonstrate that SSI-based event ticketing can enable efficient secondary market control by facilitating a practical implementation of the centralized exchange model. To generalize our results, we derive design principles for the efficient, reliable, and privacy-oriented ticket and identity verification and the use of revocation registries.

## Introduction

“On the Internet, nobody knows you’re a dog.” Since the publication of this famous cartoon by Peter Steiner (1993), the Internet has evolved and has revolutionized our lives. Still, existing solutions for digital identity verification are not satisfactory for both users and service providers (Bonneau et al., 2012; Maler & Reed, 2008; Sedlmeir et al., 2021; Smith & McKeen, 2011). The absence of a secure and reliable identity layer on the Internet affects the ticketing industry in particular. An average of 40% of ticketing portals’ traffic is caused by bots (Imperva, 2019) that create fake identities to acquire tickets and then resell them for a profit. This behavior also known as scalping, implies that persons sometimes cannot purchase tickets at all or only at much higher prices (Glaap & Heilgenberg, 2019). Also, tickets on the secondary market are sometimes offered several times, and there is no way for fans to check their validity (Waterson, 2016). This exposes users to the risk of paying for an invalid or a counterfeit ticket (Regner et al., 2019).

To control these secondary markets and protect customers, several methods have been proposed. By identifying the user at the time of purchase, ticket limits could be enforced. However, owing to the aforementioned lack of a universal digital identity layer, identity verification via isolated solutions such as eID or VideoIdent is costly for ticket portals and inconvenient for users, which is why they are usually not implemented in today’s solutions (Ehrlich et al., 2021). The more common way is to print visitors’ names on tickets (*identity-binding*) and to verify their identity at the venue’s entrance (*identity control*). However, performing analogue identity checks is time-consuming and costly owing to the additional human resources required (Waterson, 2016). Thus, other approaches apply dynamic QR codes that require the corresponding account login data (sometimes even tied to a visitor’s SIM card) to present a valid ticket at the venue: Since the QR code changes every few minutes, the ticket cannot be shared with others before the event starts (Hooking, 2019). Yet, to bypass these systems, ticket bots can create several accounts and transmit their account login data or SIM card instead of the ticket (GUTS Tickets, 2018).

To improve ticket ownership verification and secondary market control, blockchain technology has been suggested (Cha et al., 2018; Li et al., 2019; Regner et al., 2019; Tackmann, 2017). In connection with automated and rules-based transaction processing using smart contracts and non-fungible tokens (NFTs), event organizers can transparently record ticket ownership and define rules and price limits for secondary ticket market transactions (Regner et al., 2019). Yet this approach is hard to align with data protection regulations, such as the GDPR, particularly regarding the “right to be forgotten.” (Regner et al., 2019; Rieger et al., 2021; Sedlmeir et al., 2022). Also, creating a blockchain account comes with almost no costs, which is why – like the previous centralized systems – blockchain-based ticket systems still require identity-binding and control to prevent scalpers from circumventing the system (Corsi et al., 2019; Regner et al., 2019). In sum, the key challenge of the mentioned approaches is the weak binding of users to their ticket, as current identity-binding solutions – such as a user account or a SIM card in the case of a centralized ticket issuer-based solution or the blockchain account in the case of decentralized blockchain-based solutions – can be simply transferred without incurring significant costs.

Recently, a new paradigm, self-sovereign identity (SSI), for end-users’ digital identity management, has gained considerable momentum, likely also owing to blockchain technology’s popularity. Although blockchain technology is not strictly needed for SSI, several SSI projects use a blockchain as a publicly shared and immutable registry for trusted organizations (Sedlmeir et al., 2021). In the case of SSI, users store their identity-related documents in so-called digital wallet apps on their smartphones (Avellaneda et al., 2019). Different credentials can be stored and presented in combination through these identity wallets, for instance, a digital ID card, a digital vaccination certificate, and a digital ticket (Sedlmeir et al., 2021).

Using SSI-based identity verification for event ticketing systems is a promising approach to strongly binding tickets to visitors, enabling secondary market control reliably, efficiently, and at low cost. Soltani and Nguyen (2018) presented a novel SSI-based eKYC onboarding design and evaluated their solution against Allen’s principles of SSI (Allen, 2016). Compared to the architecture presented by Soltani and Nguyen that builds on one very specific technology stack, namely Hyperledger Indy, Schlatt et al. (2021) emphasize the degrees of freedom in blockchain-based SSI from a technical perspective, such as what data needs to be stored on a blockchain, also regarding nascent standards that are being actively developed by the World Wide Web Consortium (W3C), and take this degree of freedom into discussions with experts. Liu et al. (2020) identified 12 design patterns for blockchain-based SSI, addressing key management, decentralized identifier management, and credential design. Yet, SSI-based solutions do not necessarily need to be based on blockchain. For instance, Alpár et al. (2017) introduced the IRMA project, representing a solution that implements the principles of SSI without using a blockchain in its technology stack (Nauta & Joosten, 2019). While the general implementation of SSI is expected to be similar in other application domains, the examined cases only cover the use and transmission of a single credential. However, in many other domains, multiple credentials need to be verified simultaneously (iTICKET, 2021). For instance, in the case of event ticketing, this could comprise the presentation of an ID card with a high level of assurance and an event ticket at the venue’s entrance, potentially supplemented by a Covid-19 vaccination certificate.

Consequently, we explore the use of SSI and its implications for the event ticketing market in general and secondary market control in particular, but also related settings that require the verification of multiple credentials at the same time. We apply a rigorous design science research (DSR) approach following Peffers et al. (2007) to develop and evaluate an SSI-based event ticketing framework incorporating existing theoretical knowledge through a literature review as well as practitioners’ perspectives through eight semi-structured interviews with experts. By instantiating our framework in a Proof of Concept (PoC), we demonstrate our approach’s feasibility and evaluate its fitness to solve event ticketing-related problems (Hevner et al., 2004; March & Smith, 1995). To elevate our SSI artifact for more abstract and generalizable theoretical discussion, we capture the design knowledge embedded implicitly in our artifact and derive nascent design principles. Thus, we uncover valuable insights for digital identity management solutions in event ticketing and similar contexts that require efficient, privacy-oriented, and reliable identity verification.

The remainder of this paper proceeds as follows: Sect. 2 sets the theoretical foundations for event ticketing, secondary market control, and SSI. In Sect. 3, we introduce our research method. In Sect. 4, we derive design objectives for an event ticketing solution with secondary market control. We then present the SSI-based framework, including a PoC implementation, as our design artifacts in Sect. 5. Section 6 describes the evaluation of our artifacts, followed by Sect. 7, in which we summarize practical implications and elevate our research for theoretical discussion by deriving design principles. In Sect. 8, we conclude and identify limitations and avenues for further research.

## Background

### Event ticketing and secondary market control

A ticket can be defined as “a contract between a user and a service provider. If the visitor demonstrates his [or her] ownership of the ticket, he [or she] obtains the right to use the service under its terms and conditions” (Mut Puigserver et al., 2012, p. 3). One can distinguish between traditional paper tickets, electronic tickets that are delivered in digital form but can still be printed out, and digital tickets, which can only be used in digital form (e.g., dynamic QR codes) (Payeras-Capellà et al., 2017). The primary event ticketing market usually consists of at least three stakeholder types: visitors, ticket issuers, and event organizers (Chaumette et al., 2012).

Yet users occasionally want to resell their tickets. These activities take place in the secondary ticket market (The Australian Government the Treasury, 2017). While event organizers usually don’t intend to ban resales, as they wish to maximize fan attendance and give customers who cannot attend the opportunity to recoup their money by reselling their tickets (U.S. GAO, 2018), they lose their influence over ticket pricing in the secondary market (Waterson, 2016). The stakeholders’ objectives differ, dividing the secondary ticket market into two segments: the regular ticket reselling market and the scalping market (The Australian Government the Treasury, 2017). So-called scalpers buy tickets on the primary ticket market not with the plan of attending the event but to resell them in the secondary ticket market at a higher price (Segrave, 2006). Scalping also undermines event organizers’ efforts to offer tickets below market prices to make them accessible to certain fan groups (Schneiderman, 2016; U.S. GAO, 2018).

Scalpers often gain a competitive advantage by using bots, which can automatically create a large number of accounts and can swiftly carry out many purchasing processes (Courty, 2019; Waterson, 2016). According to a Ticketmaster study, 60% of the most desirable tickets from some shows are bought by bots (NYT, 2019). Another frequently mentioned problem associated with buying secondary market tickets is ticket fraud. Since buyers mostly cannot verify a ticket’s authenticity, ownership, and integrity, they run the risk of buying a counterfeit ticket. Visitors are often not even aware that they are buying a speculative ticket or that they are buying on the secondary market (U.S. GAO, 2018). While ticket scalping and bot activities can to some extent be countered by strategies like dynamic pricing (Waterson, 2016), event organizers often cannot achieve many of their objectives simultaneously, such as maximized attendance, generating additional revenues, or increasing fan satisfaction through affordable prices (Courty, 2017). Enforcing price caps, for example by voiding tickets that are sold on secondary markets at inflated prices, represents an alternative solution approach to prevent ticket scalping and bot activities. However, enforcing these price caps involves significant expenditure of resources and is usually not realized rigorously (U.S. GAO, 2018). Besides, compliance costs arise for secondary market providers, who need to monitor the tickets’ face values permanently and adapt their website accordingly (The Australian Government the Treasury, 2017).

Instead of fighting resale activities in the secondary market, more and more event organizers have decided to cooperate with secondary market platforms. Thus, the boundaries between the primary and the secondary markets are becoming increasingly blurred. So-called sponsored resale marketplaces offer customers opportunities to safely buy and sell secondary market tickets, because the ticket issuer invalidates the original ticket and guarantees the new ticket’s validity. Further, a ticket issuer can set a price range for the ticket resale on these sponsored resale markets (Courty, 2017). Nonetheless, sponsored resale markets alone cannot fully solve problems such as scalping or ticket fraud, since tickets can still be sold on other marketplaces at any price (Courty, 2019). Schneiderman (2016) identified the lack of identification of visitors as a key reason for scalpers’ undermining intentions to provide accessible ticket prices, specifically when they use bots. Thus, identity-binding is the core of Courty’s (2019) four conditions for secondary market control:

- The current legitimate owner needs to be recorded in a ledger.
- Ticket owners must be refunded when they no longer need their tickets.
- Returned tickets are randomly reallocated to previously unserved fans to ensure that scalpers cannot bypass the price limits set on the system/central exchange by receiving side-payments from ticket buyers.
- Identity checks are necessary at admission to ensure that tickets aren’t used by anyone other than the ticket owner.

This approach is called the *centralized exchange model*, since identity-binding at the time of ticket issuance and identity verification when visiting an event ensure that tickets can only be resold via a centralized exchange. Thus, this model mitigates scalping and ticket fraud activities while still allowing customers to sell tickets they no longer need.

Courty (2019) showed that the centralized exchange model can improve welfare and therefore dominate an open resale market, especially in markets with much scalping activity. However, this is not a one-size-fits-all approach owing to the significant differences between events concerning the extent of underpricing or different legal circumstances (Schneiderman, 2016). Event organizers’ pricing directly affects the secondary market by influencing resales’ profitability and thus the volume of tickets resold in the secondary market (The Australian Government the Treasury, 2017). The centralized exchange model represents a promising solution and is already applied when the significant identity verification effort is justified owing to a high level of secondary market and scalping activities, for instance, in the case of the Glastonbury Festival (Waterson, 2016). Yet, existing solutions for performing the necessary identity checks are costly and time-consuming, which is why most events don’t impose these additional controls (Regner et al., 2019; Waterson, 2016).

### Self-sovereign identity

**Fig. 1:**
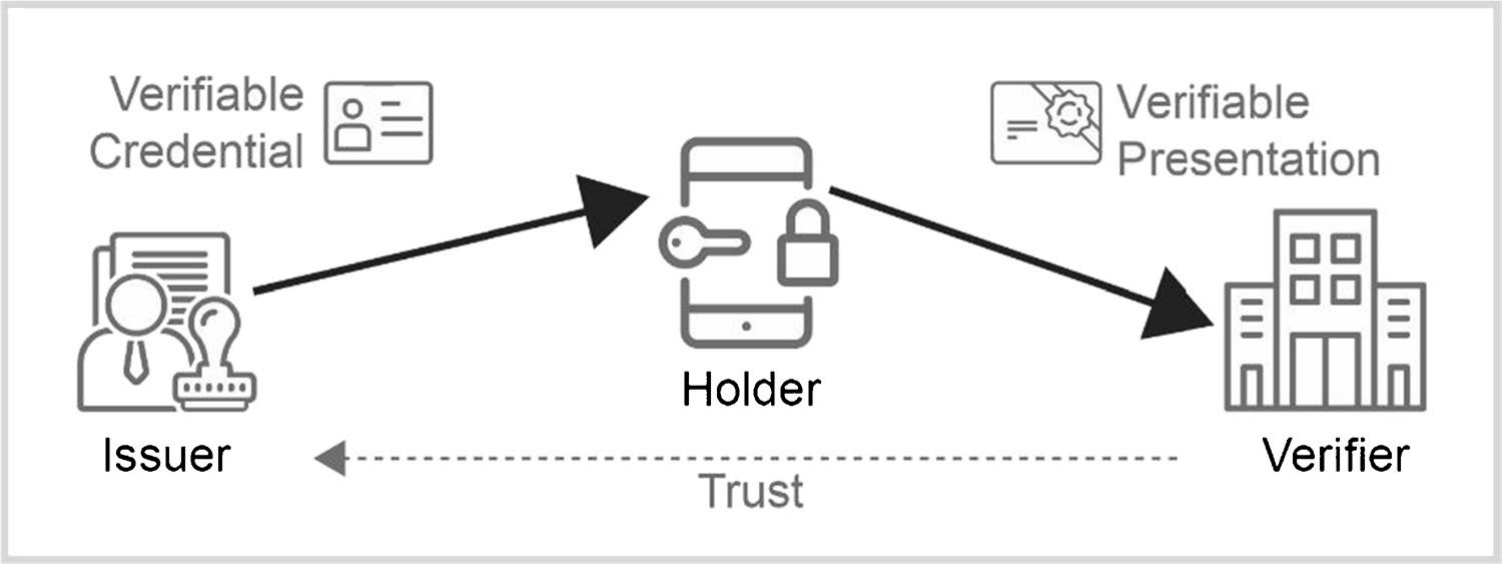
Essential roles and information flows of SSI-based identity management

SSI can be considered a paradigm shift in digital identity management, empowering users to self-manage their identities and providing them with password-less login and digital representations of many verifiable documents. It allows for independently managing and selectively sharing identity data without being limited to a single domain or use case (Wang & Filippi, 2020). SSI represents physical documents, such as standardized ID cards or access badges, that are made tamper-proof with watermarks or seals through digitally signed data objects called verifiable credentials (VCs). VCs make claims about an entity – for instance, regarding attributes (e.g., name, age), relationships (e.g., mother, daughter), or entitlements (e.g., memberships, legal status) – cryptographically provable (Preukschat & Reed, 2021; Sporny et al., 2021). No third party is needed for storing and transferring the information, allowing for the confidential sharing of verifiable personal information in bilateral interactions (Schlatt et al., 2021) (Fig. 1).

Credential holders store and manage their VCs in a software application called an *identity wallet* or a *digital wallet*, typically on their mobile phones (Lesavre, 2020). To prove certain claims, holders transmit verifiable presentations (VPs) to relying parties (*verifiers*). In such a VP, holders present proofs about the information requested by the verifier that are derived from one or more VCs to the verifier (Sporny et al., 2021). This can be done by presenting the VCs themselves, together with a proof of ownership through signing a random challenge with a secret key associated with a public binding key referenced in the VC. More privacy-preserving approaches apply cryptographic zero-knowledge proofs (ZKPs) (Preukschat & Reed, 2021) that avoid the need for highly correlatable identifiers and allow for selective disclosure (Hardman, 2020). The additional use of ZKPs for improved data minimization is already supported by some projects that provide solutions for implementing SSI, such as IRMA or Hyperledger Aries.

Thus, SSI uses public key cryptography to verify the integrity and authenticity of credentials without the need to interact with the issuer (Ehrlich et al., 2021). However, a trust relationship between a verifier and the credential issuer must first be established (Mühle et al., 2018). Some projects also use publicly available infrastructures such as blockchains as a technical backbone for managing trust relationships by recording institutions’ public keys (Ehrlich et al., 2021; Schlatt et al., 2021). Moreover, to avoid the need for communication with the credential issuer or another dedicated third party when the verifier wants to check VCs’ revocation status during a VP, revocation information is often published on a blockchain-based public registry. In some approaches, the revocation status is referenced through VCs’ unique serial numbers revealed in a VP, so the relying party can look it up during a VP. However, since a VC’s revocation status can be sensitive and personally identifiable, there are privacy-oriented revocation registries where only a cryptographic accumulator is published that allows holders to create proofs of inclusion/exclusion without revealing their VC’s serial number (Schlatt et al., 2021).

The SSI paradigm is typically not understood as replacing existing government-issued eIDs. Such an eID also plays a key role for SSI, since it is one of the foundational digital documents issued by a highly trusted institution. However, unlike existing eID approaches, SSI’s scope extends beyond a single and highly regulated identity document and seeks to use open standards to enable an ecosystem of various credential types that can be managed together in one of many interoperable digital wallets (Sedlmeir et al., 2021). Several governmental and corporate initiatives are already exploring SSI’s benefits, and undertakings are under way to combine SSI with existing trust infrastructures such as the electronic Identification, Authentication and Trust Services (eIDAS) regulation (Ehrlich et al., 2021; European Commission, 2019). SSI is also being considered for the EUid initiative, which aims to provide European citizens with interoperable digital wallets and to force large businesses to provide interfaces (European Commission, 2020).

Since SSI builds on open-source standards and users control their data without any lock-in effect, users can lever their VCs for various use cases. In particular, it is no longer necessary for customers to manually fill out forms that require their master data such as their names, address, and banking information (Wagner et al., 2018). Further, by using VCs, high trust in the transmitted data can be achieved immediately and fully automatically (Preukschat & Reed, 2021). SSI can also help to protect data. Since the identity information is stored by the user, data honey pots – which aggregate identity information and are a popular target for attacks – can be avoided (Schlatt et al., 2021). With selective disclosure and ZKPs, only the minimum of data required for a use case can be transmitted, so SSI also resonates with the GDPR’s privacy requirements of privacy by design and by default (Der et al., 2017).

Yet some general challenges must be considered. First, the responsibility for managing and securing keys and creating backups is entirely up to the users (Lesavre, 2020). Second, identity theft is a particular challenge for user-centric identity management systems such as SSI (Lesavre, 2020). In the event of identity theft, it must be ensured that attackers cannot gain access to the identity wallet. The biometric unlocking of digital wallets is already a valuable option (Preukschat & Reed, 2021). Besides, when credentials (e.g. a VC tickets) can only be used in combination with a government ID card that is stored in the same wallet, it may be sufficient to revoke and re-issue the ID card to prevent an attacker from using the stolen credentials. Third, the ability to share or sell credentials must be prevented to ensure that users can be identified with high level of assurance (Camenisch & Lysyanskaya, 2001). A strong bond between users and their credentials can be achieved in several ways, such as using secure hardware, biometrics, cryptography, and incentives. As an example, Othman and Callahan (2018) introduced the Horcrux Protocol, a method for decentralized biometric-based SSI. Fourth, regulatory requirements pose a challenge for SSI, since there is often no clear guidance on the legal implications of digital signatures (Wagner et al., 2018).

## Method

We follow a DSR approach to conceptualize and evaluate a novel SSI-based event ticketing framework and to derive generalizable design knowledge in the form of design principles. DSR seeks to solve business problems by creating innovative IT artifacts through a build-and-evaluate process, with the created artifacts’ utility ensured by applying rigorous methods (Hevner et al., 2004; March & Smith, 1995; Nunamaker & Chen, 1990; Walls et al., 1992). The build process includes all activities to create something innovative, while the evaluation aims to get feedback and to better understand the problem at hand, allowing for the artifact’s improvement (Markus et al., 2002). Key results of the DSR approach are the creation of an innovative artifact, scientific abstraction, and learning (Beck et al., 2013).

We structured our research by following the frequently used and widely accepted DSR process of Peffers et al. (2007). Since we followed a problem-centered approach, we first became aware of the problem at hand (1). Our examination of the event ticketing literature and systems revealed several challenges, including scalping and the use of ticket bots, fraud activities, a lack of transparency when purchasing on the secondary market, and challenges implementing Courty’s centralized exchange model. Based on the acquired understanding of the problem and existing requirements for event ticketing systems and secondary market control (Courty, 2019; Mut Puigserver et al., 2012), we then derive design objectives to overcome the identified challenges (2). This approach has five main objectives and several detailed sub-requirements, which serve as a basis for creating and evaluating our artifact. In the next step (3), we designed our SSI-based event ticketing framework and instantiated a PoC based on related work on event ticketing and seminal work on the foundations and applications of SSI. We then demonstrated the artifact (4) to experts to get their feedback and to iteratively improve our artifact. Next, we provided a criteria-based evaluation (5) of our artifact along the design objectives based on the experts’ feedback and the technical design. To ensure the practical fit, we focused on understandability, applicability, and functionality (Sonnenberg & vom Brocke, 2012). The artifact’s evaluation is a crucial step in DSR to provide evidence that the artifact fulfils its purpose and therefore generates utility in its application environment. Further, using rigorous methods are key during evaluation so as to ensure knowledge outcome quality (Hevner & Chatterjee, 2010; Venable et al., 2016).

We used qualitative interviews as the primary method to generate rich data (Schultze & Avital, 2011). As a first step, we carefully selected experts who have long dealt with SSI or event ticketing during their daily work and can therefore evaluate our artifact (Morse, 1991). By choosing experts working in different domains (ticket issuing, event organization, SSI) and companies related to our research, we aim to provide a diverse perspective on the ticketing process and on emerging technical solutions in the realm of event ticketing. Through our choice of experts, we can approach the complexities of the research topic from both the domain side as well as the technical side. By incorporating well-established research on the consumer perspective on event-ticketing, we refrain from interviewing end users. However, we acknowledge that generic usability studies of SSI are an interesting future research endeavor (Dunphy & Petitcolas, 2018). Table 1 provides an overview over the experts and their backgrounds. On average, the interviews lasted 55 minutes (shortest: 41 min, longest: 86 min).

**Table 1:** Overview of the interviewed experts

Expertise	ID	Brief description	Position
Event organization	1	Event manager specialized in event ticketing	Head of ticketing and marketing, event organizer
Event organization	2	Event manager specializing in event ticketing	Head of ticketing, professional sport team
Event organization	3	Media marketing expert with a focus on new media and ticketing	Head of new media and ticketing, concert organizer
SSI and blockchain	4	Consultant specializing in decentralized identity management	SSI project manager, R&D department
SSI and blockchain	5	Blockchain architect specializing in decentralized identity management	Blockchain architect, blockchain startup
Ticket issuance	6	Founder and CEO of a blockchain-based ticketing startup with a background in asset management and financial trading	CEO, blockchain and identity-based ticketing startup
Ticket issuance	7	Event ticketing expert with a focus on digital ticket innovations and secondary market solutions	Team lead ticketing, professional sport team
Ticket issuance	8	Digital innovations expert working on the digital transformation of ticketing and merchandise	Head of digital strategy and innovation, professional sport team

We transcribed and subsequently analyzed the interviews using grounded theory analysis techniques (Corbin & Strauss, 2015). Therefore, we inductively formed categories and subcategories solely based on the available data. We coded the data using an initial open coding round (Saldaña, 2009), assigning a conceptual label to logically connected text sections that summarize what is induced by the text (Kuckartz, 2018).

Coding round 1 resulted in 36 categories and 424 subcategories. In coding round 2, we applied axial coding. A permanent reflection of the data served to identify relationships between individual open codes and to summarize the identified categories on a more abstract level (Charmaz, 2006; Corbin & Strauss, 2015). To elevate the design knowledge implicit in our artifact to more abstract and generalizable knowledge, we derived nascent design principles for the efficient, reliable, and privacy-oriented ticket and identity verification from the codes. As the final step in our applied research process, we communicate our results (6) in this paper.

## Design objectives

Based on a literature analysis, we derived five design objectives and associated requirements for our SSI-based event ticketing framework (see Table 2). In the literature analysis, we examined requirements for event ticketing and secondary market control as well as SSI particularities. We will discuss the design objectives and their fulfillment in depth in the evaluation section.

**Table 2:** Design objectives for the event ticketing framework

Design objectives		Description
1. Secondary market control	*1.1 Tickets are bound to visitors*	When a ticket is purchased, it must be bound to that visitor. In the case of a ticket resale, the ticket issuer must invalidate the old ticket and must create a new ticket bound to the new owner (Courty, 2019; Regner et al., 2019)
	*1.2 Reliable and efficient entrance identification*	To effectively prevent unauthorized ticket resales, event organizers must verify that the ticket owner’s identity matches the name recorded on the ticket before entry to the venue is permitted (Courty, 2019). Thus, identity verification at the entry must be reliable (Schneiderman, 2016). Attackers should be unable to impersonate other visitors through eavesdropping and replay attacks (Ekberg & Tamrakar, 2012). Also, the verification of identity documents, tickets, and other credentials at the venue’s entrance must be efficient, to save time and costs (Waterson, 2016)
	*1.3 Random reallocation of returned tickets*	Returned tickets should be randomly reallocated. This prevents scalpers from selling tickets in the regular secondary market and receiving side-payments from ticket buyers (Courty, 2019)
2. Bot prevention	*Effective limitation of ticket purchases per person*	The number of tickets that a single entity can buy should be efficiently limited to a predefined number (Waterson, 2016). A fair distribution of tickets among regular fans should be enabled. The use of ticket bots to circumvent these limits must therefore be prevented (Courty, 2019)
3. Ticket validation	*3.1 Authenticity and integrity*	Both visitors and the event organizer must be able to verify whether a ticket has been issued by an authorized issuer (*authenticity*) and whether it has been modified afterward (*integrity*). This should also apply to secondary market tickets (Mut Puigserver et al., 2012)
	*3.2 Ownership verification*	Visitors, especially those who buy their ticket in the secondary market, must always be able to verify the ownership of their tickets (Regner et al., 2019). Event organizers must also be able to verify ticket ownership at the venue’s entrance (Courty, 2019)
4. Privacy	*Confidentiality and compliance with data protection regulations*	Visitors should be able to purchase and redeem tickets while minimizing the amount of personal information that needs to be disclosed (Vives-Guasch et al., 2012). Data protection regulations (e.g., the GDPR) provide strict legal rules on storing and processing personal data and represent a constraint that needs to be met. They require privacy by default and design. The processing and storage of personal data must be restricted to the intended purpose and stakeholders, and records need to be deleted at visitors’ request (Rieger et al., 2019)
5. User-friendliness	*User-friendly access to tickets and events*	User-friendliness is a key requirement for any ticketing system, including the given hardware requirements, which should not make the event inaccessible to certain visitor groups. Alternative solutions should also be available if visitors cannot meet the given hardware requirements, for instance, if some visitors don’t have Internet-capable smartphones (Mut Puigserver et al., 2012; Regner et al., 2019)

## Self-sovereign identity-based event ticketing framework

Our framework is a concrete instantiation of the centralized exchange model according to Courty (2019). It is based on SSI to identify visitors, both when buying a ticket and when entering the venue. A detailed technical description of SSI-based interactions and workflows can be found, for instance, in Schlatt et al. (2021). The implemented PoC serves to demonstrate the SSI-based aspects of our event ticketing framework and to illustrate its differences to existing systems. The PoC is implemented using Trinsic, a platform that supports developing SSI use cases and that provides an SSI wallet that is interoperable with other SSI wallets such as the Lissi wallet or the ID wallet supported by Germany’s government.

**Fig. 2:**
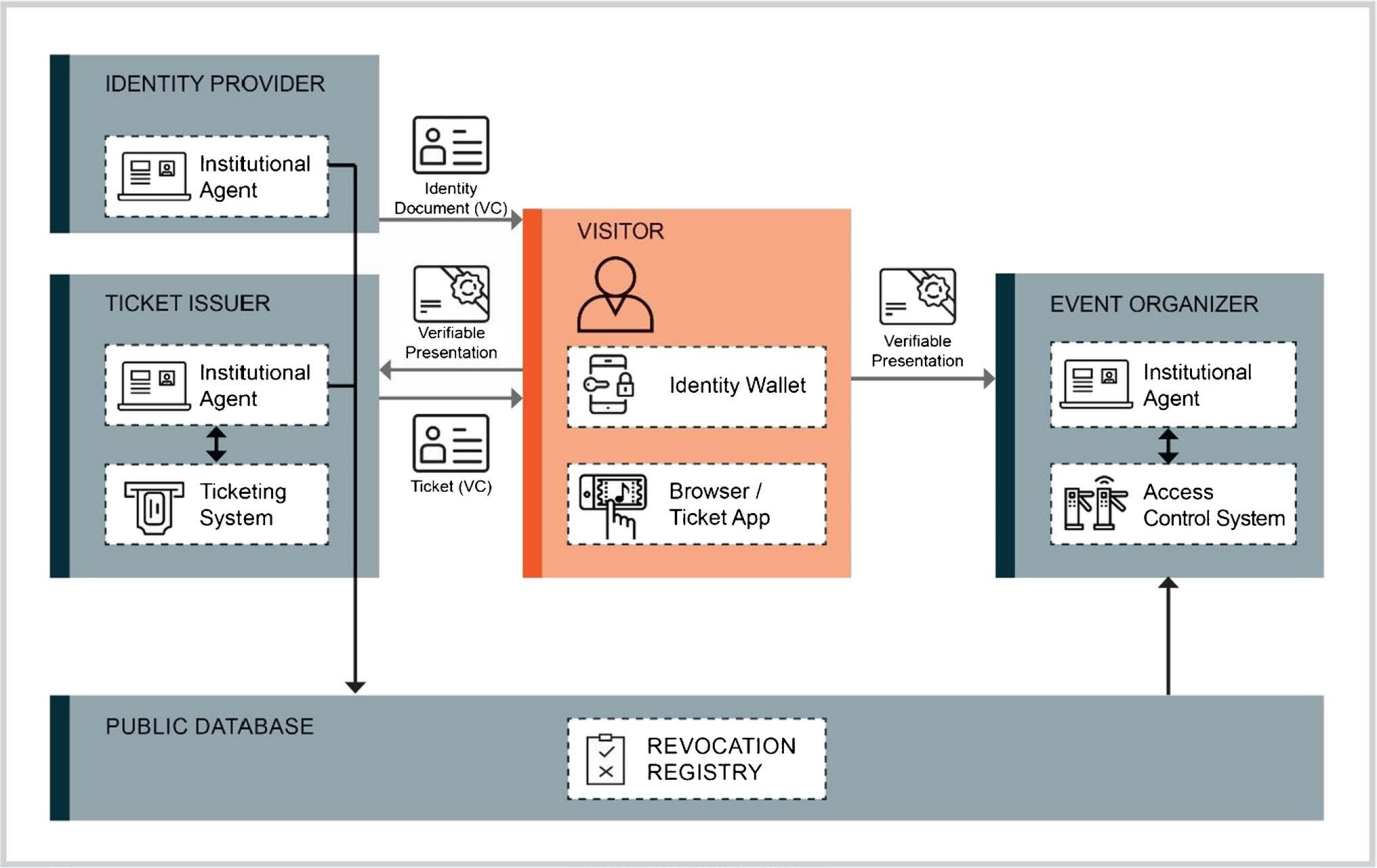
SSI-based event ticketing architecture

Figure 2 illustrates our SSI-based event ticketing architecture, comprising identity providers, visitors, ticket issuers, and event organizers. In this context, ticket portals and identity providers act as issuers of VCs, visitors as credential holders, and event organizers as verifiers. A public database is used to record revocation registries. Using such a public revocation registry allows issuers to revoke, i.e., invalidate prior to expiry, issued credentials. Event organizers are responsible for managing events and controlling visitors at the venue’s entrance and can issue tickets themselves or can outsource this to specialized ticket issuing services. If the sale is made via an external ticket issuer, the event organizer and the ticket issuer must first negotiate the available tickets, primary and secondary market ticket prices, and discounts for each event. They must also define a governance framework in advance to define which identity providers and which VC types the venue should accept for user identification, such as ID cards, driver’s licenses, or health insurance cards. Besides, they need to define whether further resale associates are allowed to sell tickets and get access to the ticket issuers’ database.

Visitors’ identity wallets are the architecture’s core. Visitors manage identity-related documents in their digital wallet. Tasks like cryptographic key management and backups are fully managed in the background. When receiving a proof request for identity information, the wallet automatically searches for VCs that satisfy the proof request’s requirements and prompts the visitor for confirmation to create and respond with a VP containing the requested information. In the context of the Covid-19 pandemic, a digital vaccination certificate could also be stored as a VC, simultaneously enabling identity, ticket, and vaccination status verification and ‘with one click.’ To search for upcoming events and to purchase or sell appropriate tickets, ticket buyers can use either a browser or a dedicated app provided by the ticket issuer. However, tickets are stored as VCs in the visitor’s identity wallet.

**Fig. 3:**
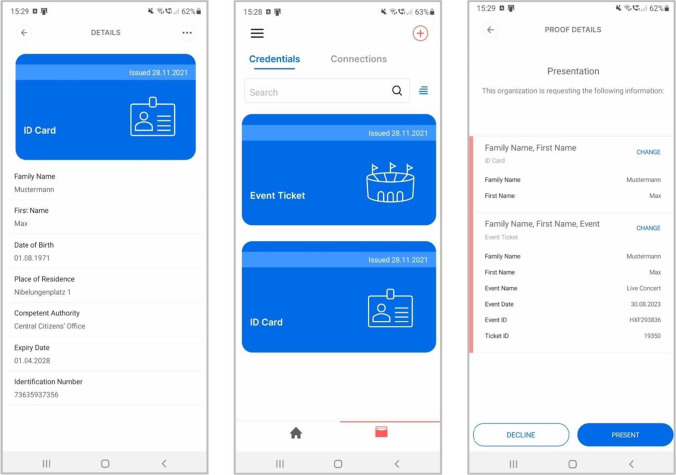
Identity wallet – left: ID card credential offer; center: credential overview; right: event proof request

In the SSI-based framework, visitors undergo four high-level process steps. Identity provisioning (1) only needs to be carried out if the visitors don’t yet possess suitable identity credentials for user registration. It comprises the visitor’s *base identity*, for which data such as name or date of birth need to be provided (see Fig. 3, on the left). Governmental authorities mostly take the role of the identity providers for these document types such as ID cards or driver’s licenses which provide a high level of assurance. Since official SSI-based identity documents are not yet widely available, a specialized KYC provider can also perform identity provisioning to ensure the availability of identity credentials (Ostern & Riedel, 2021). Thus, visitors can receive a VC after proving their identity to a KYC provider. Depending on the event, other identity-related information can also be necessary, such as vaccination status or a club membership credential (iTICKET, 2021). Identity providers and event organizers use so-called institutional agents – a software specializing in the issuance and revocation of VCs and for the requesting and verification of VPs (Schlatt et al., 2021).

For user registration (2), the ticket issuer requests one or more credentials from visitors to uniquely identify them. To this end, visitors receive a corresponding proof request on their identity wallet, which contains the required attributes and specifications regarding accepted VCs from certain credential issuers. The identity wallet automatically creates a VP in the proof format requested by the ticket issuer. The only manual step for visitors is the authorizing of the release of the VP to the ticket issuer. Based on publicly available data, the ticket issuers can verify the transmitted credentials’ validity and revocation status (Schlatt et al., 2021). After their VP has been verified, visitors are successfully registered. The entire process can be highly automated and is completed in seconds. A separate ticketing system is used to manage individual events and available tickets.

Via a website or app provided by the ticket issuer, users can purchase tickets (3) after successful identification and authorization. The necessary payment information could also be transmitted via VCs to increase the payment process’ user-friendliness and security (Association of German Banks, 2021). Finally, the ticket issuer sends the actual digital ticket to the visitor. The tickets are not delivered as PDF files but in the form of VCs and, thus, as digital tickets, allowing for integrity, authenticity, and ownership verification. The verifiable tickets are sent directly to the visitor’s wallet. If the visitors have bought several tickets, a download link can be sent to their companions. In case the companions don’t yet have an identity wallet and necessary credentials, they must first undergo the identity provisioning (1) and user registration (2) steps to receive their ticket. When their companions are successfully registered, they receive a ticket offer in their identity wallet. Thus, both buyers and their companions have personalized tickets and the credentials necessary for identity verification (see Fig. 3, center).

To enter the venue (4), a visitor scans a QR code at the event access control system. The QR code contains a proof request for a ticket and identity VCs as well as a random challenge to prevent replay attacks (Tackmann, 2017). The visitor’s identity wallet then automatically creates a VP that meets all the necessary requirements (see Fig. 3, on the right). The wallet sends the requested proof directly to the event organizer’s institutional agent whose service endpoint is also specified in the proof request, using a mobile Internet connection or WLAN. The event organizer can thus verify the validity (integrity, authenticity, and non-revoked state) of both the ticket and the identity document, ensuring that they refer to the same person, for instance by comparing cryptographically binding information or first and last name. The institutional agent finally sends the verification’s result to the event access control system, which grants the visitor access to the event if the verification is successful.

**Fig. 4:**
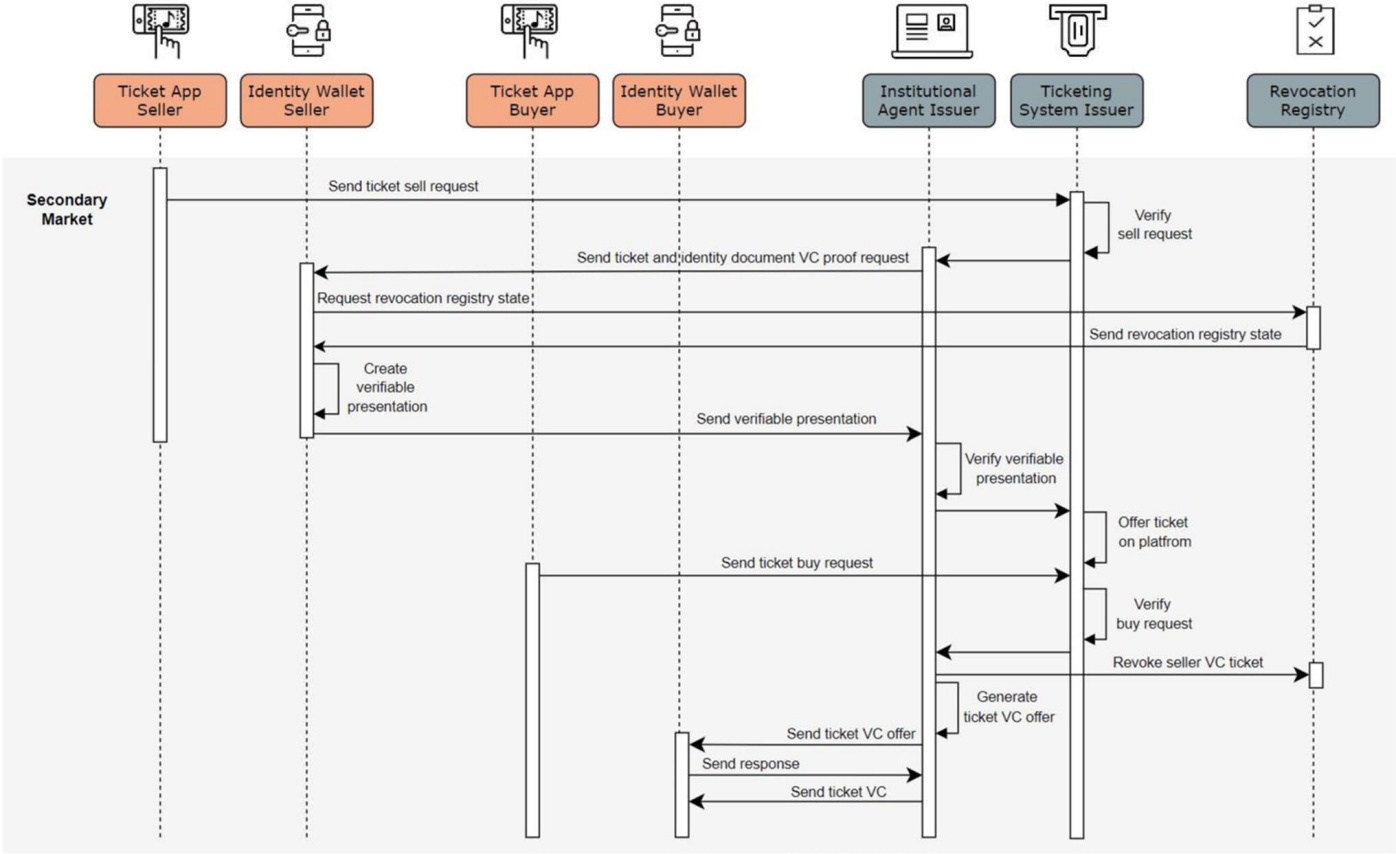
UML diagram: secondary market transaction

The procedure for buying a secondary market ticket is similar to purchasing a primary market ticket. Users first must undergo the (1) identity provisioning and (2) user registration steps. Whenever a customer wants to sell a ticket, the ticket issuer needs to verify the ticket’s authenticity and ownership and subsequently offers the ticket on the ticket platform. The ticket issuer revokes the original ticket once another user has bought the ticket. To do so, an update of the revocation registry ensures that the old ticket is no longer valid (Schlatt et al., 2021). Finally, the ticket issuer creates a new ticket that is bound and sent to the new owner. Thus, the ticket issuer ensures that there is only one valid ticket in a “chain of resales” at any given time (see Fig. 4).

## Evaluation

We conducted interviews with experts to evaluate our design and PoC implementation (see Sect. 3). Based on their feedback and the lessons from the PoC, we adapted our artifact. For instance, we added vaccination certificates as additional VCs that can be requested when buying the ticket or entering the venue. We will now consolidate our findings by providing a summative, criteria-based analysis, assessing the specified design objectives’ fulfillment.

### DO1: Secondary market control

The ticket issuer binds every ticket to its current owner (R1.1). To do so, each visitor must first register with the issuer to receive a personalized ticket in the form of a VC, which can later be presented together with other VCs such as digital ID cards in a single VP. This also applies to tickets resold in the secondary market, whereby the ticket issuer invalidates the original ticket through revocation and the new owner receives a new ticket. At the venue’s entrance, both the verifiable ticket and a verifiable identity credential are requested. As the requested VCs are not self-attested but issued by a trusted thirty party, SSI offers reliable identity verification (experts 1, 4, 6). Thus, the de facto level of assurance can be increased by demanding a government-issued identification document or a credit card, since passing them on is associated with increased risk (experts 1, 4, 6). Further, the experts listed several technical protection measures to strengthen the bond, such as challenge-response procedures, as applied in our artifact (experts 1, 4–7). SSI-based user identification also allows for reliable *and* efficient user identification (R1.2), since data verification occurs automatically in the background and requires only a single confirmation in the user’s digital wallet (experts 1, 3, 4). This eliminates the need for manual inspection at the venue’s entrance, saving time and personnel (experts 1, 3, 6–7). Yet, the experts also identified some additional expenses, such as employee training and improving the existing communication infrastructure at the venue (experts 1, 2, 5). While the digital distribution and verification of tickets are in principle also possible without the use of SSI, SSI provides standardized digital wallet and institutional agent components, and supports the digital verification of both tickets and identities in a single step and also allows for inheriting a high level of assurance from other VCs (experts 2–3, 6).

As a result of requirements 1.1 and 1.2, tickets cannot be transferred arbitrarily, but can only be sold in the official secondary market with involvement of the ticket issuer, where they are randomly reallocated (R1.3) to prevent side-payments from happening. This way, users can get compensation for their tickets, but cannot switch companions (experts 1, 3, 6–7). Thus, the experts emphasized that it’s impractical to implement a random reallocation of tickets for all event types. Instead, this should be decided depending on the individual event and the expected black-market activity (experts 1–3, 6–7). According to expert 3, event organizers often face significant overheads concerning events in high demand owing to the increased black-market and ticket fraud activities, which is why our SSI-based solution adds significant value to these event types. Nonetheless, SSI can also be useful for other events to ensure a consistent user experience and to verify additional credentials such as Covid-19 vaccination certificates.

### DO2: Bot prevention

Our artifact provides two avenues for ticket bot prevention (R2): First, by unambiguously identifying ticket buyers, for instance through their names and date of birth or a serial number on their government-issued ID card. In this scenario, the ticket issuer can check whether a ticket buyer already has opened an account, since state authorities ensure that each citizen has at most one valid digital ID card at any time (Berg et al., 2018; Wang & Filippi, 2020). Thus, ticket bots cannot create many accounts, since they lack the necessary credentials (experts 4–5). While this approach helps to prevent a crowding out of regular fans on the primary ticket market, eliminating ticket bots does not completely prevent ticket scalping activities, since one of the main causes of scalping is ticket underpricing. As soon as the demand for tickets exceeds the supply due to underpricing and inelastic supply of tickets, an arbitrage opportunity arises for scalpers by reselling the tickets on the secondary market (Schroeder et al. 2012).

Second, by implementing our approach for secondary market control, it’s no longer profitable for scalpers to operate ticket bots because they can barely resell tickets a higher price (expert 6). While the prevention of ticket bots through unambiguously identifying ticket buyers thus represents an intermediate solution compared to a free resale market by reducing comparative advantages, the variant presented in this paper enables an almost complete prevention of both ticket scalping and bot activities but does not allow users to gift their tickets to others.

### DO3: Ticket validation

Tickets, issued as VC such as in our artifact, are tamper-proof and authentic through the issuer’s digital signature. Thus, both visitors and the event organizer can verify the ticket’s authenticity and integrity at any time (Mühle et al., 2018) (R3.1). Tickets are also bound to the visitor’s identity credentials cryptographically or through highly correlating attributes such as name and date of birth. Thus, a transfer of ownership is only possible through resale in the official secondary ticket market or by both transmitting the ticket and the associated identity credential when reselling the ticket in the black-market (experts 6–7). Using government-issued IDs with a high level of assurance that are typically hardware-bound to a device, and in general valuable identity documents that are costly to pass on (e.g., a credit card), the second option is far less attractive than passing only an isolated account or a SIM card. For tickets bought in the official secondary markets, visitors can always verify their ownership (R3.2). However, expert 6 pointed out that the visitors need to trust the ticket issuers, which could take away their tickets again, for instance to serve other quotas. Expert 7 noted that tickets are only withdrawn in the case of fraudulent behaviors. Also, withdrawing tickets could damage the ticket issuer’s reputation.

### DO4: Privacy

Concerning privacy protection, a conflict arises between ticket personalization, which is necessary to bind tickets to visitors and thus to control the secondary market, and the implementation of an anonymous event ticketing system (experts 4, 6–7). Visitors have to provide personal data for ticket personalization, which can be accessed by the ticket issuer (expert 3) and optionally used for bot prevention purposes (experts 3, 5–7). Thus, tickets cannot be bought completely anonymously. However, ZKP technology, as used in our artifact, has the potential to somewhat eliminate this tradeoff between privacy and unambiguous identification (expert 5). For instance, visitors can submit a ZKP that they hold a valid (non-expired, non-revoked), government-issued ID card without transmitting their de facto ID card in plain text and provide binding information that the issuer cannot use to identify the user yet integrate in the ticket to strongly bind it to the ID card. Thus, SSI technology opens new possibilities for privacy protection compliance with data protection regulations (R4). Yet, to offer anonymity, such approaches would likely need to be supplemented by opportunities for legally compliant, anonymous payments – as discussed for instance in the context of the digital euro and that could also be achieved with SSI and ZKPs (Gross et al., 2021).

In any case, visitors manage their data independently with their identity wallet, giving them full control over their digital identities (Lyons et al., 2019). Only the ticket issuer and event organizer with a need to request and process some visitor data receive personal information, and visitors get an overview in their digital wallet over whom they have shared which data with. As soon as the reason for the data being stored no longer exists, these data can be deleted (expert 4). Also, no personal data is stored at other parties or on a blockchain. Thus, compliance with the GDPR’s fundamental objectives can likely be achieved (expert 8).

### DO5: User-friendliness

The use of SSI can positively impact privacy and security, but also increases personal responsibility for visitors and raises new questions regarding user-friendly ticket and event access (R5) that need to be answered by SSI wallets. Since our solution did not build a new user-facing app but relied on digital wallets that will likely be regularly applied for many identification, authentication, and authorization purposes, the consistent user experience can likely increase user acceptance and confidence (expert 4).

As specified in our event ticketing framework, every event attendant must first register with the ticket issuer to receive a personalized ticket in the form of a VC, which implies that ticket buyers and all their companions need to be clearly identified by the issuer (experts 1, 4–5, 7). Alternatively, the companions’ identity could be verified first at the venue’s entrance; however, this complicates the entry process. For instance, using this approach, the entitlement to any ticket discounts can only be checked at this point using the corresponding credentials. Another alternative is omitting the personalization of the companions’ tickets, such that only the ticket purchaser’s identity is initially recorded and verified at the entrance. However, this approach limits scalping prevention and involves complexities, for instance if the ticket buyer cannot attend the event (experts 7–8).

Our interviews revealed that the goal of low hardware requirements for SSI-based event ticketing is hard to achieve. To receive a digital ticket in the form of a VC, visitors need to own a mobile device. This can cause problems, especially for older visitors (experts 1–3, 6–7) or in the presence of hardware defects or empty batteries (Preece & Easton, 2019). As an alternative, users should thus have the opportunity to receive personalized paper tickets. However, in this case, the identity must be checked manually at the entrance against a valid ID document. Users also need an Internet connection at the venue’s entrance to retrieve the current state of the revocation registry to prove non-revocation (experts 3, 5, 7); however, it is conceivable that, in the future, SSI wallets can receive the revocation state for which they need to prove non-revocation bilaterally from the verifier, i.e., the event organizer’s institutional agent.

## Discussion

The experts broadly confirmed that our artifact can significantly improve the event ticketing domain, especially the prevention of ticket scalping, fraud, and bots. Further, several aspects we investigated can be translated to similar settings that simultaneously require efficient credential verification, privacy protection, and identity-binding with high levels of assurance. Thus, as a result of our DSR approach to rigorously build and evaluate a novel IT artifact, incorporating both existing kernel theories on SSI and event ticketing as well as the profound knowledge of experts working in these fields, we gained valuable insights. To capture the design knowledge embedded implicitly in our artifacts and render it accessible, we analyzed the identity management related codes from the evaluation and put them in context to derive nascent design principles. Our design principles, therefore, allow us to abstract from the concrete instantiation of the Proof of Concept that represents one flavor of SSI and a small share of digital identity management solutions. Following a structured approach based on Gregor et al. (2020), we defined the context for every design principle individually, acknowledging that our design principles’ generalizability is limited to their various boundary conditions. We further outlined the aim, implementer, and user, the individual mechanism, and the underlying rationale to ensure the design principles’ feasibility, applicability, and reusability (Gregor et al., 2020).

### DP1: Facilitate digital credential-bundling and verification

#### Aim, implementer, user

By facilitating digital credential bundling and verification, organizations can automatically verify different identity information types issued by different organizations across domains without the need for manual inspections or several user-sided process steps, increasing process efficiency for both users and service providers. Further, users can repeatedly lever their existing credentials, which renders cumbersome registration and identification steps obsolete by transmitting the requested information in digital form and gives them familiarity with the process.

#### Mechanism and rationale

A key objective of identity management is to facilitate access to services in one or several application domains (Ferdous et al., 2019). Yet because most identity management systems are isolated, credentials from one application domain cannot be used in other domains, since interoperable standards and trust frameworks across domains are often missing. Our research suggested that by using user-centric identity management systems and following interoperable standards, the digitization of identity-related credentials in SSI provides the necessary foundation for efficient and user-friendly identification processes. It enables the use of VCs in different contexts, such that data transmission and verification can be done automatically in the background (experts 1, 3–4). This also eliminates the need for manual displaying and inspection, saving time and personnel (experts 1, 3). These benefits are amplified in situations where identity claims from different credentials need to be verified simultaneously, such as identity, address, and payment information, health information (e.g., vaccination status), and authorizations (e.g., a driver’s license). By personalizing and issuing these documents as VCs, event organizers and other credential verifiers such as public transportation operators can verify VCs’ validity and whether they were all issued to the same user. Thus, the benefits of user-centric approaches such as SSI increase as the credential ecosystem grows. Our results suggest that governments’ support for SSI benefits both organizations and citizens. The SSI-based event ticketing artifact illustrates an example where different credentials (e.g., ID cards, vaccination passports, and tickets) are bundled within one identity wallet and are verified simultaneously at the venue’s entrance to speed up the verification and increase efficiency (experts 1, 3, 5–6).

#### Context

While our empirical investigation was limited to event ticketing, it can be abstracted to more general settings where identity claims from different credentials and domains need to be verified at the same time, both online and physically.

### DP2: Bind credentials to users using existing credentials with a high level of assurance

#### Aim, implementer, user

Our SSI-based artifact represents a concrete implementation of the centralized exchange model (Courty, 2019). Thus, the artifact must ensure that a ticket can only be used by its associated owner. The same holds true for many other use cases, where credential verifiers need to be sure that a presented credential (e.g., a vaccination certificate) is in fact bound to the person presenting it.

#### Mechanism and rationale

Although VC-based user identification (issued and signed by trusted third parties) can be considered more reliable than self-attested claims, the level of assurance depends on various factors. Several attack vectors must be considered, concerning man-in-the-middle attacks, device thefts, and voluntary disclosure to third parties (expert 4). These attacks are possible owing to users’ missing or insufficient binding to their credentials (experts 1, 4–5). The stronger a user’s binding to their credentials, the less likely it is that third parties may use them. Thus, passing on one’s identity credentials (and authenticators) must be associated with considerable cost (experts 6–7). In light of this, solutions based solely on blockchain technology for assuring the ownership of event tickets – as proposed by several authors (Li et al., 2019; Regner et al., 2019; Tackmann, 2017) – seem insufficient. Here, the users are identified by their private key, and the ticket is bound to it. Yet the blockchain solution can be bypassed by simply transmitting the private key (something one knows), which involves almost no costs.

In SSI, the associated cost can be increased by additionally requesting official ID cards, credit cards, or other VCs for which passing them on would mean losing one’s central identity representation or taking a significant risk of being held accountable for actions (expert 1, 4, 6–7). In event ticketing, it is sufficient if these associated costs of passing on credentials to ticket buyers are in the order of magnitude of the ticket’s value. Thus, credentials with a high level of assurance can act as a *golden source of identification*. This can give “all-or-nothing non-transferability” (Camenisch & Lysyanskaya, 2001) to other credentials within one’s identity wallet, which can inherit the high level of assurance of government-issued IDs. To prove that these credentials have been issued to the same person, it suffices to verify whether an ID card’s strongly correlatable attributes like name and date of birth match the attributes on the credentials. By cryptographically binding credentials to an ID card or other VCs with a high level of assurance (e.g., through a blinded link secret) (Schlatt et al., 2021), this high level of assurance can even be inherited without having to present sensitive information. Other technical measures, as discussed in the evaluation section, can further strengthen the bond between the users and their credentials (experts 1, 4–6).

#### Context

Ensuring a strong bond between a user and their credentials is relevant for several use cases beyond event ticketing, for instance in vaccination certificates, identity and access management, e-prescriptions, or various mobility services. The use of SSI provides VCs with a high level of assurance and privacy-protecting technologies such as ZKPs opens a spectrum of options for defining the required levels of assurance and privacy protection.

### DP3: Use public and privacy-preserving revocation registries to manage resale activities

#### Aim, implementer, user

Controlling secondary markets requires binding tickets strictly to visitors (see DP2) and efficiently verifying their identity and ticket ownership at the venue’s entrance (see DP1) (Courty, 2019). As tickets shall still be transferable, the current legitimate owner needs to be recorded in a database to distinguish between valid and invalid tickets. The recording of ticket ownerships on VC-based tickets in combination with public yet privacy-preserving revocation registries creates an interesting variant of Courty’s “ledger,” avoiding the need for storing ticket owners in a proprietary, siloed database with access control while also allowing event organizers to use standardized infrastructure for VC verification.

#### Mechanism and rationale

While Courty listed the storage of the current ticket owner in a ledger as a condition for the centralized exchange model, the ledger’s implementation is left open. The two prevailing approaches we presented in the introduction are a ledger maintained by the ticket issuer and read access for the event organizer, and a blockchain where ownership of the ticket in the form of an NFT is recorded transparently. A proprietary, private ledger may be challenging from an interoperability and access control perspective, while the NFT-based approach on a public ledger bears privacy challenges. Using a public but privacy-oriented revocation registry and zero-knowledge proofs of in-/exclusion as facilitated by several prevailing digital wallets allows for the use of proofs of ownership without involving the ticket issuer and without compromising users’ privacy, taking the best from the two approaches. While a public and privacy-oriented ledger for NFT-based tickets, similar for example to Zcash that also utilizes zero-knowledge proofs, would also be feasible, they require special integration, whereas via using SSI’s revocation registries, the ticket issuer can leverage a mature, interoperable solution.

#### Context

Privacy-preserving, public revocation registries are used in many SSI implementations to enable the confidential verification of a credential’s revocation status (Preukschat & Reed, 2021; Schlatt et al., 2021). The verification of tickets and their revocation status works exactly the same way and uses the same infrastructure as for other identity documents, such as VC-based ID cards or Covid-19 vaccination certificates. This reduces the cost of developing systems and facilitates their integration into other infrastructures such as public transportation, since direct communication between the ticket issuer and the verifier in question is not necessary for credential verification.

### Opportunities and challenges of using SSI when implementing the centralized exchange model

As a result of our SSI-based event ticketing framework using the centralized exchange model, ticket owners cannot simply pass their ticket on to a third party but must request the ticket transfer from the ticket issuer. This approach enables (1) ticket issuers, holders, and event organizers to verify a ticket’s ownership. Thus, only the ticket’s legitimate owner can enter the venue, preventing ticket fraud activities where a single ticket is sold several times. Also, by additionally implementing a random reallocation of tickets to prevent side-payments, this approach ensures (2) that the price and resale restrictions in this platform can barely be circumvented (Courty, 2019). Thus, ticket scalping and black-market activities can be effectively prevented*. “As a result, everyone profits, but no one enriches themselves”* (expert 3). Since tickets cannot be resold at a profit or only with a small margin, it is also (3) no longer profitable to operate ticket bots to get a competitive advantage and buy large numbers of tickets, increasing the chance of regular ticket buyers receiving a ticket (experts 6–8).

While our SSI-based event ticketing framework benefits visitors (reduced ticket fraud and scalping, user-friendly identity verification), ticket issuers (control over secondary market transactions), and event organizers (efficient entrance verification, increased fan satisfaction), some challenges remain. To purchase SSI-based event tickets, users must first possess suitable identity credentials. This corresponds to the “crossing the chasm challenge” observed by Schlatt et al. (2021). Once the users are equipped with a digital wallet and foundational credentials as planned for instance with the EUid, the onboarding process at the ticket issuer can be fully automated. Besides governmental organizations, also other organizations such as banks could act as trustworthy identity providers, which could also allow them to improve their own onboarding and authentication processes and create new revenue streams (Birch, 2021; Schlatt et al., 2021). A seamless onboarding process on its own may even be attractive enough for ticket issuers to integrate SSI technology (expert 1). As a further challenge resulting from our approach, capping ticket resale prices at the original face value may result in a net-loss for customers if ticket fees are not refundable. Yet, this could be avoided to some extent by allowing resales within a fixed range around the original price. As an additional challenge, tickets cannot be transferred arbitrarily or gifted to others but can only be sold in the official ticket secondary market, where they need to be randomly reallocated to completely prevent scalping. This means that visitors can get compensation, but cannot change their companions after purchasing their tickets, which spectators could disvalue (experts 1, 3, 7). Thus, the experts emphasized that it’s impractical to implement a random reallocation of tickets for all event types. Instead, this should be decided depending on the individual event and the expected black-market activity (experts 1–3, 6–7). Event organizers often face significant overheads concerning events in high demand owing to the increased black-market and ticket fraud activities, which can be eliminated by following our proposed design (expert 3).

## Conclusion

While several papers and projects have explored the use of blockchain for event ticketing systems (Aventus, 2020; GET, 2017; Li et al., 2019; Regner et al., 2019), none have focused on the integration of digital identity management to solve existing problems such as scalping and ticket fraud, even though reliable identity verification is a key requirement for solving these issues (Courty, 2019). Also, these approaches pose unresolved privacy problems. The need for efficient and reliable identity binding also became apparent during the expert interviews, demonstrating that existing solutions (both traditional centralized approaches and more recent blockchain-based approaches using NFTs) are not sufficient. Thus, there is a lack of knowledge on designing and evaluating a solution that solves these challenges. To address this research gap, we followed a design science research approach based on Peffers et al. (2007). We build on different research streams such as SSI-based designs (Hoess et al., 2022; Schlatt et al., 2021; Soltani & Nguyen, 2018), well-known design requirements for event ticketing systems (Mut Puigserver et al., 2012; Regner et al., 2019; Vives-Guasch et al., 2012), as well as specific design requirements for secondary market control (Courty, 2019) to design and evaluate an SSI-based event ticketing framework and to gain insights on a higher level of theoretical abstraction.

Our contributions to the existing body of knowledge are threefold. First, we have provided a novel, SSI-based event ticketing approach. By implementing and evaluating a PoC, we have also demonstrated the feasibility of an SSI-based event ticketing approach and its fitness to solve event ticketing-related problems such as scalping and ticket fraud (Drechsler & Hevner, 2018). We found that SSI allows for reliable and efficient user identification, representing an effective solution to implement the centralized exchange model proposed by Courty (2019). We also found that issuing digital tickets as VCs and using SSI’s privacy-oriented revocation capabilities for resale activities in the official secondary market has considerable advantages for all stakeholders. Second, design principles are still rare in the innovative field of SSI-based applications. By providing novel design principles, we have uncovered valuable insights for digital identity management based solutions in the context of event ticketing and similar contexts that require efficient, privacy-oriented, and reliable identity verification. Thus, deriving novel design principles allowed us to elevate our IT artifact for more abstract and generalizable theoretical discussion (Gregor & Hevner, 2013). Third, we have revealed theoretical insights regarding the merits of SSI for event ticketing. By proposing revocation registries, we have extended Courty’s model with an additional way of implementing the binding of visitors to their tickets, as opposed to storing the current holders explicitly in a centralized or decentralized ledger. Further, the SSI-based approach offers high flexibility since additional credentials (e.g., vaccination certificates) can be requested without running into efficiency or privacy issues.

Our event ticketing framework holds valuable insights for practitioners. By transparently developing and rigorously evaluating the artifact, we have provided useful findings regarding the implications of individual design choices. Further, we illustrated opportunities and challenges resulting from the use of SSI for event ticketing systems, which managers can include in their decision-making process. We found that using SSI can be beneficial, especially at events in high demand with a resulting increase in scalping and fraud activities.

Our research has limitations, which can stimulate further research. The SSI-based event ticketing framework focuses on the centralized exchange model to solve existing challenges such as scalping. Yet further research is necessary to explore the feasibility and the consequences of SSI-based ticketing in the context of an open resale market. Furthermore, we led most interviews with senior executives from German-speaking regions, which might stir a regional and elite bias in the insights. Interviewing experts in the domain of SSI with a blockchain background might also stir a technological bias. Therefore, future research should regard perspectives from practitioners and users with more diverse cultural and technical backgrounds to test the propositions’ validity. The current revision of the eIDAS regulation and the introduction of an EUID wallet (European Commission, 2022) as well as the development of digital wallets in the private sector, for example by companies like Google (Phillips, 2022), present promising opportunities for this purpose. Also, we evaluated the framework primarily from an ex-ante perspective. While we gained first evidence of practical feasibility and utility, an evaluation in a practical large-scale event ticketing system is necessary to confirm our results. This will also allow to study the user perspective on SSI in event ticketing and similar contexts, thereby providing an insight which is currently lacking most research. As a further limitation, SSI is still in its infancy. Consequently, both technological aspects (Schellinger et al., 2022), usability aspects (Sartor et al., 2022) and the ecosystem around SSI (Laatikainen et al., 2021; Schlatt et al., 2021) need to mature for large-scale use. A sophisticated ID solution is necessary to bundle credentials and support privacy-preserving revocation and, thus, realize the results presented in this paper. Yet, besides SSI, other solutions that provide digital identity wallets for users and exhibit similar characteristics (Phillips, 2022) could also be used. Our design principles, e.g., with regard to revocation, might thus generalize beyond the scope of SSI, which however needs to be tested.

Based on our work, researchers can follow various promising research avenues, particularly concerning finding solutions to the identified challenges such as minimizing the hardware requirements. Technologically oriented researchers could explore the integration of NFC or other bilateral communication technologies such as Bluetooth into the SSI stack, as this approach provides an attractive opportunity to conduct VPs at access terminals and in other situations without a guaranteed Internet connection, in both a user-friendly and secure way or explore alternative digital identity management schemes that offer credential bundling and public revocation registries. Also, the privacy-oriented use of biometrics is a potential research direction to link the digital and the physical worlds. While this could help bind users more strongly to their credentials, it challenges privacy. Economically oriented researchers could explore in detail the savings potential of digital identity wallet based identity verification compared to conventional methods, both at the point of purchase and at the entrance to an event. Further, we recommend exploring the benefits of practical digital identity verification in other sectors where the combination of multiple credentials or invalidation based on a public registry can improve existing processes.

## References

[CR1] Allen, C. (2016). *The path to self-sovereign identity*. http://www.lifewithalacrity.com/2016/04/the-path-to-self-soverereign-identity.html

[CR2] Alpár, G., van den Broek, F., Hampiholi, B., Jacobs, B., Lueks, W., & Ringers, S. (2017). *IRMA : practical decentralized and privacy-friendly identity management using smartphones*. https://www.semanticscholar.org/paper/IRMA-%3A-practical-%2C-decentralized-and-identity-using-Alp%C3%A1r-Broek/4bfefe33c5e143bb1cd4f3aca96539cb7289483b

[CR3] Association of German Banks. (2021). *Digital identities – steps on the path to an ID ecosystem*. https://en.bankenverband.de/newsroom/comments/digital-identities-steps-path-id-ecosystem/#2

[CR4] Avellaneda O, Bachmann A, Barbir A, Brenan J, Dingle P, Duffy KH, Maler E, Reed D, Sporny M (2019). Decentralized identity: where did it come from and where is it going?. IEEE Communications Standards Magazine.

[CR5] Aventus. (2020). *Aventus white paper: the ultimate blockchain guide*. https://www.aventus.io/wp-content/uploads/2020/03/The-Aventus-Whitepaper-2020-.pdf

[CR6] Beck, R., Weber, S., & Gregory, R. W. (2013). Theory-generating design science research. *Information Systems Frontiers,**15*(4), 637–651. 10.1007/s10796-012-9342-4

[CR7] Berg, A., Berg, C., Davidson, S., & Potts, J. (2018). The institutional economics of identity. *SSRN Electronic Journal.* Advance online publication. 10.2139/ssrn.3072823

[CR8] Birch, D. (2021). *Digital identity should be a big business for banks.* Forbes. https://www.forbes.com/sites/davidbirch/2021/09/16/digital-identity-should-be-a-big-business-for-banks/?sh=14bffa2b7c3f

[CR9] Bonneau, J., Herley, C., van Oorschot, P. C., & Stajano, F. (2012, May 20–23). The quest to replace passwords: A framework for comparative evaluation of web authentication schemes. *2012 IEEE Symposium on Security and Privacy* (pp. 553–567). IEEE. 10.1109/SP.2012.44

[CR10] Camenisch, J., & Lysyanskaya, A. (2001). An efficient system for non-transferable anonymous credentials with optional anonymity revocation. In G. Goos, J. Hartmanis, J. van Leeuwen, & B. Pfitzmann (Eds.), *Lecture notes in computer science. Advances in cryptology — EUROCRYPT 2001* (Vol. 2045, pp. 93–118). Springer Berlin Heidelberg. 10.1007/3-540-44987-6_7

[CR11] Cha, S.‑C., Peng, W.‑C., Hsu, T.‑Y., Chang, C.‑L., & Li, S.‑W. A blockchain-based privacy preserving ticketing service. *IEEE 7th Global Conference 2018* (pp. 585–587). 10.1109/GCCE.2018.8574479 (Original work published 2019)

[CR12] Charmaz, K. (2006). *Constructing grounded theory: A practical guide through qualitative analysis*. Sage Publications Ltd.

[CR13] Chaumette, S., Dubernet, D., Ouoba, J., Siira, E., & Tuikka, T. (2012). Architecture and evaluation of a user-centric NFC-enabled ticketing system for small events. In J. Y. Zhang, J. Wilkiewicz, & A. Nahapetian (Eds.), *Lecture Notes of the Institute for Computer Sciences, Social Informatics and Telecommunications Engineering. Mobile Computing, Applications, and Services* (Vol. 95, pp. 137–151). Springer Berlin Heidelberg. 10.1007/978-3-642-32320-1_10

[CR14] European Commission. (2019). *eIDAS supported self-sovereign identity*. https://ec.europa.eu/futurium/en/system/files/ged/eidas_supported_ssi_may_2019_0.pdf

[CR15] European Commission. (2020). *Proposal for a European digital identity (EUid) and revision of the eIDAS regulation*. https://op.europa.eu/de/publication-detail/-/publication/35274ac3-cd1b-11ea-adf7-01aa75ed71a1

[CR16] European Commission. (2022). *Digital identity for all Europeans*. https://ec.europa.eu/info/strategy/priorities-2019-2024/europe-fit-digital-age/european-digital-identity_en

[CR17] Corbin, J. M., & Strauss, A. L. (2015). *Basics of qualitative research: techniques and procedures for developing grounded theory* (4. ed.). SAGE Publications.

[CR18] Corsi, P., Lagorio, G., & Ribaudo, M. (2019). TickEth, a ticketing system built on Ethereum. In C.-C. Hung & G. A. Papadopoulos (Eds.), *Proceedings of the 34th ACM/SIGAPP Symposium on Applied Computing - SAC '19* (pp. 409–416). ACM Press. 10.1145/3297280.3297323

[CR19] Courty P (2019). Ticket resale, bots, and the fair price ticketing curse. Journal of Cultural Economics.

[CR20] Courty, P. (2017). *Secondary ticket markets for sport events*. http://web.uvic.ca/~pcourty/HSEPascal3.0.pdf

[CR21] Der, U., Jähnichen, S., & Sürmeli, J. (2017). *Self-sovereign Identity − Opportunities and Challenges for the Digital Revolution*. https://arxiv.org/abs/1712.01767

[CR22] Drechsler, A., & Hevner, A. R. (2018). Utilizing, producing, and contributing design knowledge in DSR projects. In S. Chatterjee, K. Dutta, & R. P. Sundarraj (Eds.), *Lecture Notes in Computer Science. Designing for a Digital and Globalized World* (Vol. 10844, pp. 82–97). Springer International Publishing. 10.1007/978-3-319-91800-6_6

[CR23] Dunphy P, Petitcolas FA (2018). A first look at identity management schemes on the blockchain. IEEE Security & Privacy.

[CR24] Ehrlich T, Richter D, Meisel M, Anke J (2021). Self-Sovereign identity als Grundlage für universell einsetzbare digitale Identitäten. HMD Praxis Der Wirtschaftsinformatik.

[CR25] Ekberg, J.‑E., & Tamrakar, S. (2012). Mass transit ticketing with NFC mobile phones. In D. Hutchison, T. Kanade, J. Kittler, J. M. Kleinberg, F. Mattern, J. C. Mitchell, M. Naor, O. Nierstrasz, C. Pandu Rangan, B. Steffen, M. Sudan, D. Terzopoulos, D. Tygar, M. Y. Vardi, G. Weikum, L. Chen, M. Yung, & L. Zhu (Eds.), *Lecture Notes in Computer Science. Trusted Systems* (Vol. 7222, pp. 48–65). Springer Berlin Heidelberg. 10.1007/978-3-642-32298-3_4

[CR26] Ferdous, M. S., Chowdhury, F., & Alassafi, M. O. (2019). In search of self-sovereign identity leveraging blockchain technology. *IEEE Access,**7*, 103059–103079. 10.1109/ACCESS.2019.2931173

[CR27] GET. (2017). *Guaranteed entrance token: smart event ticketing protocol*. https://get-protocol.io/files/GET-Whitepaper-GUTS-Tickets-latest.pdf

[CR28] Glaap, R., & Heilgenberg, M.‑C. (2019). Digitales Ticketing. In L. Pöllmann & C. Herrmann (Eds.), *Der digitale Kulturbetrieb* (2018–5, pp. 127–159). Springer Fachmedien Wiesbaden. 10.1007/978-3-658-24030-1_7

[CR29] Gregor, S., Kruse, L., & Seidel, S. (2020). Research perspectives: The anatomy of a design principle. *Journal of the Association for Information Systems*, *21*, 1622–1652. 10.17705/1jais.00649

[CR30] Gregor, S., & Hevner, A. R. (2013). Positioning and presenting design science research for maximum impact. *MIS Quarterly,**37*(2), 337–355. 10.25300/MISQ/2013/37.2.01

[CR31] Gross, J., Sedlmeir, J., Babel, M., Bechtel, A., & Schellinger, B. (2021). Designing a Central Bank digital currency with support for cash-like privacy. *SSRN Electronic Journal*. 10.2139/ssrn.3891121

[CR32] GUTS Tickets. (2018). *FAQ — Can scalpers bypass the system by buying tickets on single use sim cards and selling these?* https://blog.guts.tickets/faq-can-scalpers-bypass-the-system-by-buying-tickets-on-throw-away-simcards-and-selling-these-f24e9a27e2b7

[CR33] Hardman, D. (2020). *No paradox here: ZKPs deliver Savvy trust.* https://www.evernym.com/blog/no-paradox-here-zkps-deliver-savvy-trust/

[CR34] Hevner A, March ST, Park J, Ram S (2004). Design science in information systems research. MIS Quarterly.

[CR35] Hevner, A., & Chatterjee, S. (2010). *Design research in Information systems* (Vol. 22). Springer US. 10.1007/978-1-4419-5653-8

[CR36] Hoess, A., Roth, T., Sedlmeir, J., Fridgen, G., & Rieger, A. (2022). With or without Blockchain? Towards a decentralized, SSI-based eRoaming architecture. In T. Bui (Ed.), *Proceedings of the Annual Hawaii International Conference on System Sciences, Proceedings of the 55th Hawaii International Conference on System Sciences.* Hawaii International Conference on System Sciences. 10.24251/HICSS.2022.562

[CR37] Hooking, M. (2019). *The O2 and The SSE Arena, Wembley, launch fan-first ticketing approach with AXS*. https://www.eventindustrynews.com/news/the-o2-and-the-sse-arena-wembley-launch-fan-first-ticketing-approach-with-axs

[CR38] Imperva. (2019). *How bots affect ticketing*. https://www.imperva.com/resources/resource-library/reports/how-bots-affect-ticketing/

[CR39] iTICKET. (2021). *iTICKET front foot vaccine pass pre-verification for event ticketing*. https://blog.iticket.co.nz/posts/iticket-front-foot-vaccine-pass-pre-verification-for-event-ticketing

[CR40] Kuckartz, U. (2018). *Qualitative Inhaltsanalyse. Methoden, Praxis, Computerunterstützung* (4. Auflage). *Grundlagentexte Methoden*. Beltz Juventa. http://ebooks.ciando.com/book/index.cfm?bok_id/2513416

[CR41] Laatikainen, G., Kolehmainen, T., & Abrahamsson, P. (2021). Self-sovereign identity ecosystems: benefits and challenges. *12th Scandinavian Conference on Information Systems*. https://aisel.aisnet.org/scis2021/10

[CR42] Lesavre, L. (2020). *A taxonomic approach to understanding emerging blockchain identity management systems.*10.6028/NIST.CSWP.0114202010.6028/NIST.CSWP.01142020

[CR43] Li, X., Niu, J., Gao, J., & Han, Y. (2019). Secure electronic ticketing system based on consortium blockchain. *KSII Transactions on Internet and Information Systems*, *13*(10). 10.3837/tiis.2019.10.022

[CR44] Liu, Y., Lu, Q., Paik, H.‑Y., Xu, X., Chen, S., & Zhu, L [Liming] (2020). Design pattern as a service for blockchain-based self-sovereign identity. *IEEE Software*, *37*(5), 30–36.10.1109/MS.2020.2992783

[CR45] Lyons, T., Courcelas, L., & Timsit, K. (2019). *Blockchain and digital identity*. https://www.eublockchainforum.eu/sites/default/files/report_identity_v0.9.4.pdf

[CR46] Maler E, Reed D (2008). The venn of identity: options and issues in federated identity management. IEEE Security & Privacy.

[CR47] March ST, Smith GF (1995). Design and natural science research on information technology. Decision Support Systems.

[CR48] Markus ML, Majchrzak A, Gasser L (2002). A design theory for systems that support emergent knowledge processes. MIS Quarterly.

[CR49] Morse, J. (1991). *Qualitative nursing research: a contemporary dialogue*. SAGE Publications, Inc. 10.4135/9781483349015

[CR50] Mühle, A., Grüner, A., Gayvoronskaya, T., & Meinel, C. (2018). A survey on essential components of a self-sovereign identity. *Computer Science Review,**30*, 80–86. 10.1016/j.cosrev.2018.10.002

[CR51] MutPuigserver, M., Payeras-Capellà, M. M., Ferrer-Gomila, J. L., Vives-Guasch, A., & Castellá-Roca, J. (2012). A survey of electronic ticketing applied to transport. *Computers & Security,**31*(8), 925–939. 10.1016/j.cose.2012.07.004

[CR52] Nauta, J. C., & Joosten, R. (2019). *Self-sovereign identity: A comparison of IRMA and Sovrin*. https://www.researchgate.net/publication/334458009_Self-Sovereign_Identity_A_Comparison_of_IRMA_and_Sovrin

[CR53] Nunamaker, J. F., & Chen, M. (1990, January 2). Systems development in Information systems research. *Twenty-Third Annual Hawaii International Conference on System Sciences* (pp. 631–640). IEEE Comput. Soc. Press. 10.1109/HICSS.1990.205401

[CR54] NYT. (2019). *Concert industry struggles with ‘bots’ that siphon off tickets.* The New York Times. https://www.nytimes.com/2013/05/27/business/media/bots-that-siphon-off-tickets-frustrate-concert-promoters.html

[CR55] Ostern NK, Riedel J (2021). Know-your-customer (KYC) requirements for initial coin offerings. Business & Information Systems Engineering.

[CR56] Othman AA, Callahan J (2018). The horcrux protocol: a method for decentralized biometric-based self-sovereign identity. International Joint Conference on Neural Networks (IJCNN).

[CR57] Payeras-Capellà MM, MutPuigserver M, Castellá-Roca J, Bondia-Barceló J (2017). Design and performance evaluation of two approaches to obtain anonymity in transferable electronic ticketing schemes. Mobile Networks and Applications.

[CR58] Peffers K, Tuunanen T, Rothenberger MA, Chatterjee S (2007). A design science research methodology for information systems research. Journal of Management Information Systems.

[CR59] Phillips, T. (2022). *Google launches multipurpose digital wallet with support for digital IDs, tickets and payment cards*. https://www.nfcw.com/2022/05/12/377096/google-launches-multipurpose-digital-wallet-with-support-for-digital-ids-tickets-and-payment-cards/

[CR60] Preece, J., & Easton, J. (2019). *Blockchain technology as a mechanism for digital railway ticketing.*10.13140/RG.2.2.23692.67209

[CR61] Preukschat, A., & Reed, D. (2021). *Self-sovereign identity: decentralized digital identity and verifiable credentials.*

[CR62] Regner, F., Urbach, N., & Schweizer, A. (2019). *NFTs in practice – Non-fungible tokens as core component of a blockchain-based event ticketing application*. https://www.fim-rc.de/Paperbibliothek/Veroeffentlicht/1045/wi-1045.pdf

[CR63] Rieger, A., Guggenmos, F., Lockl, J., Fridgen, G., & Urbach, N. (2019). Building a blockchain application that complies with the EU General Data Protection Regulation. *MIS Quarterly Executive,**18*(4), 263–279. 10.17705/2msqe.00020

[CR64] Rieger A, Roth T, Sedlmeir J, Fridgen G (2021). The privacy challenge in the race for digital vaccination certificates. Med.

[CR65] Saldaña, J. (2009). *The coding manual for qualitative researchers* (First published 2009). Sage. http://gbv.eblib.com/patron/FullRecord.aspx?p=585421

[CR66] Sartor, S., Sedlmeir, J., Rieger, A., & Roth, T. (2022). Love at first sight? A user experience study of self-sovereign identity Wallets. *30th European Conference on Information Systems,* Timisoara, Romania.

[CR67] Schellinger, B., Sedlmeir, J., Willburger, L., Strüker, J., & Urbach, N. (2022). *Mythbusting Self-Sovereign Identity (SSI): Diskussionspapier zu selbstbestimmten digitalen Identitäten*. https://www.fit.fraunhofer.de/content/dam/fit/de/documents/Whitepaper_Mythbusting_Self-Sovereign_Identity.pdf

[CR68] Schlatt, V., Sedlmeir, J., Feulner, S., & Urbach, N. (2021). Designing a framework for digital KYC processes built on blockchain-based self-sovereign identity. *Information & Management,**103553*. 10.1016/j.im.2021.103553

[CR69] Schneiderman, E. (2016). *What’s blocking New Yorkers from getting tickets*. https://ag.ny.gov/pdfs/Ticket_Sales_Report.pdf

[CR70] Schultze U, Avital M (2011). Designing interviews to generate rich data for information systems research. Information and Organization.

[CR71] Sedlmeir J, Smethurst R, Rieger A, Fridgen G (2021). Digital identities and verifiable credentials. Business & Information Systems Engineering.

[CR72] Sedlmeir J, Lautenschlager J, Fridgen G, Urbach N (2022). The transparency challenge of blockchain in organizations. Electronic Markets.

[CR73] Segrave, K. (2006). *Ticket scalping: an American history, 1850–2005*. McFarland & Company Inc. Publishers. http://gbv.eblib.com/patron/FullRecord.aspx?p=1784029

[CR74] Smith, H. A., & McKeen, J. D. (2011). The identity management challenge. *Communications of the Association for Information Systems*, *28.*10.17705/1CAIS.02811

[CR75] Soltani, R., & Nguyen, U. T. (2018). A new approach to client onboarding using self-sovereign identity and distributed ledger. *2018 IEEE International Conference on Internet of Things (iThings) and IEEE Green Computing and Communications (GreenCom) and IEEE Cyber, Physical and Social Computing (CPSCom) and IEEE Smart Data (SmartData)* (pp. 1129–1136). IEEE. 10.1109/Cybermatics_2018.2018.00205

[CR76] Sonnenberg, C., & vom Brocke, J. (2012). Evaluations in the science of the artificial – Reconsidering the build-evaluate pattern in design science research. In D. Hutchison, T. Kanade, J. Kittler, F. Mattern, J. C. Mitchell, M. Naor, O. Nierstrasz, C. Pandu Rangan, B. Steffen, M. Sudan, D. Tygar, M. Y. Vardi, G. Weikum, K. Peffers, M. Rothenberger, & B. Kuechler (Eds.), *Lecture Notes in Computer Science. Design Science Research in Information Systems. Advances in Theory and Practice* (Vol. 7286, pp. 381–397). Springer Berlin Heidelberg. 10.1007/978-3-642-29863-9_28

[CR77] Sporny, M., Longley, D., & Chadwick, D. (2021). *Verifiable credentials data model 1.0: expressing verifiable information on the Web*. https://w3c.github.io/vc-data-model/

[CR78] Steiner, P. (1993, July 5). On the Internet, nobody knows you are a dog. *The New Yorker,* 1993.

[CR79] Tackmann, B. (2017). Secure event tickets on a blockchain. In J. Garcia-Alfaro, G. Navarro-Arribas, H. Hartenstein, & J. Herrera-Joancomartí (Eds.), *Data privacy management, cryptocurrencies and blockchain technology* (pp. 437–444). Springer International Publishing.

[CR80] The Australian Government the Treasury. (2017). *Ticket reselling in Australia*. https://consult.treasury.gov.au/small-business-and-consumer-division/ticket-reselling-in-australia/supporting_documents/cs2017t234743.pdf

[CR81] U.S. GAO. (2018). *Event ticket sales: market characteristics and consumer protection issues*. https://www.gao.gov/assets/700/691247.pdf

[CR82] Venable J, Pries-Heje J, Baskerville R (2016). FEDS: A framework for evaluation in design science research. European Journal of Information Systems.

[CR83] Vives-Guasch, A., Payeras-Capellà, M. M., Mut Puigserver, M., Castellá-Roca, J., & Ferrer-Gomila, J. L. (2012). A secure E-ticketing scheme for mobile devices with near field communication (NFC) that includes exculpability and reusability. *IEICE Transactions on Information and Systems*, *E95-D*(1), 78–93. 10.1587/transinf.E95.D.78

[CR84] Wagner, K., Nèmethi, B., Renieris, E., Lang, P., Brunet, E., & Holst, E. (2018). *Self-sovereign identity: a position paper on blockchain enabled identity and the road ahead*. https://www.bundesblock.de/wp-content/uploads/2019/01/ssi-paper.pdf

[CR85] Walls JG, Widmeyer GR, El Sawy OA (1992). Building an information system design theory for vigilant EIS. Information Systems Research.

[CR86] Wang, F., & Filippi, P. de (2020). Self-sovereign identity in a globalized world: Credentials-based identity systems as a driver for economic inclusion. *Frontiers in Blockchain*, *2*, Article 28. 10.3389/fbloc.2019.00028

[CR87] Waterson, M. (2016). *Independent review of consumer protection measures concerning online secondary ticketing facilities*. https://bit.ly/2wLvnrB

